# Increasing psychological flexibility is associated with positive therapy outcomes following a transdiagnostic ACT treatment

**DOI:** 10.3389/fpsyt.2024.1403718

**Published:** 2024-07-15

**Authors:** Ronja Rutschmann, Nina Romanczuk-Seiferth, Andrew Gloster, Christoph Richter

**Affiliations:** ^1^ Clinic for Psychiatry, Psychotherapy and Psychosomatics, Vivantes Klinikum Kaulsdorf, Berlin, Germany; ^2^ Department of Psychiatry and Neurosciences, Charité – Universitätsmedizin Berlin, Corporate Member of Freie Universität Berlin and Humboldt-Universität zu Berlin, Berlin, Germany; ^3^ Department of Psychology, MSB Medical School Berlin, Berlin, Germany; ^4^ Faculty of Behavioral Sciences and Psychology, University of Lucerne, Lucerne, Switzerland

**Keywords:** Acceptance and Commitment Therapy, transdiagnostic approach, unified treatment, psychological flexibility, AAQ-II

## Abstract

**Objectives:**

Increasing psychological flexibility is considered an important mechanism of change in psychotherapy across diagnoses. In particular, Acceptance and Commitment Therapy (ACT) primarily aims at increasing psychological flexibility in order to live a more fulfilling and meaningful life. The purpose of this study is to examine 1) how psychological flexibility changes during an ACT-based treatment in a transdiagnostic day hospital and 2) how this change is related to changes in symptomatology, quality of life, and general level of functioning.

**Methods:**

90 patients of a psychiatric day hospital participated in the study. Psychological flexibility, symptomatology, and quality of life were assessed at three measurement time points (admission, discharge, and 3-month follow-up). The level of functioning was assessed at admission and discharge. Differences in psychological flexibility were tested via two-sided paired samples *t*-tests. Correlations of residualized change scores were calculated to detect associations between changes in psychological flexibility and other outcomes.

**Results:**

Psychological flexibility increased significantly from pre-treatment to post-treatment (*d* = .43, *p* <.001) and from pre-treatment to follow-up (*d* = .54, *p* <.001). This change was significantly correlated to a decrease in symptomatology (*r* = .60 –.83, *p* <.001) and an increase in most dimensions of quality of life (*r* = -.43 – -.75, *p* <.001) and general level of functioning (*r* =-.34, *p* = .003).

**Discussion:**

This study adds further evidence for psychological flexibility as a transdiagnostic process variable of successful psychotherapy. Limitations are discussed.

## 1 Introduction

Traditionally, most clinical research on behavioral therapy focusses on specific mental disorders rather than transdiagnostic treatments (1, 2). While this arguably makes the studies more comparable, it also creates problems when transferring the results into the real world. First of all, the focus on disorder-specific treatments stands in striking contradiction to high rates of comorbidity. A nationally representative study in Germany, for example, found comorbidity rates of over 40 percent among individuals with psychiatric diagnoses (3). A second issue that arises with diagnosis-specific treatments, is the restricted efficiency. Mono-diagnostic treatments require clinicians to use different manuals or techniques for every disorder, making their training and preparation more costly and time-consuming (4, 5). This could also be a reason why clinicians seldom use treatment manuals, despite their proven effectiveness (6). Also, providing disorder-specific inpatient units or group therapy may not be economically feasible everywhere. Clinics may not be able to fund manuals and training for all the different diagnoses. Additionally, smaller clinics, in particular, may not have enough patients with each diagnosis to plan diagnosis-specific groups or units (4). Third, it has been increasingly observed in recent years that the mechanisms behind different mental disorders and their treatments are often very similar (1, 2).

One hypothesized transdiagnostic mechanism is psychological flexibility. Already in the 1940s, researchers found that mental health was related to flexible and contextual behavior (7, 8). In the last decades, the concept of psychological flexibility gained more attention with the rise of Acceptance and Commitment Therapy (ACT), a third-wave behavioral therapy approach. Psychological flexibility according to ACT can be defined as “the tendency to respond to situations in ways that facilitate valued goal pursuit” (9, p. 2), which includes being in touch with the present moment and the feelings it comes with, without fighting them unnecessarily (10). Psychological flexibility becomes especially important in challenging situations (9), and is closely linked to resilience (11).

ACT considers its counter pole, psychological inflexibility, or the “inability to persist or change in the service of long term valued ends” (12, p. 6), as a major source of psychopathology. This is true regardless of the diagnosis. For example, avoidance of fear or pain and non-value-orientated behavior leads to suffering, whether the diagnosis is anxiety disorder, post-traumatic stress disorder, depression, or somatoform disorder.

The hypothesis of psychological inflexibility as an important factor of psychopathology is supported by existing research: Metanalyses indicate moderate to large correlations between psychological inflexibility and different measures of psychopathological symptoms, stress, pain, and reduced quality of life (12–14). Therefore, ACT has the primary goal of promoting psychological flexibility in order to be able to live a full, vibrant and meaningful life (10, 12). Unlike other therapeutic approaches, this approach considers symptom reduction only as a by-product. The main goal remains the improvement of the subjective quality of life (15).

ACT seeks to promote psychological flexibility through six core processes: being present, acceptance of (unpleasant) inner events, defusion from unhelpful thoughts, understanding the self as context (rather than concept), being aware of one’s values and following them through committed action (12). ACT assumes that just as psychological inflexibility leads to suffering, regardless of diagnosis, so the promotion of psychological flexibility leads to improvement, regardless of diagnosis. This makes ACT a genuinely transdiagnostic therapeutic approach (16). Additionally, ACT has a strong focus on therapy processes rather than only outcomes (17).

The efficacy of ACT has been demonstrated in more than one thousand RCT studies (18) and several meta-analyses (cf. 19). Corresponding to the underlying theory, mediation analyses report increasing psychological flexibility as a mediator or process variable of ACT treatment effects, such as increases in quality of life and decreases in symptoms (20–22). However, although ACT sees itself as a transdiagnostic approach of psychotherapy, most studies continue to examine primarily disorder-specific or non-clinical contexts. Only recently, the first studies on ACT in transdiagnostic clinical settings have been published (23–25). All of them reported significant improvements in symptoms. Gloster et al. (25) have found psychological flexibility to moderate the association between stress and symptoms as well as disability. Morgan et al. (23) reported a significant increase in psychological flexibility during the treatment. Ohse et al. (24) found a significant association between the increases of psychological flexibility and the decrease of symptoms during treatment. These studies contribute important new insights into the use of ACT in transdiagnostic clinical settings and the role of psychological flexibility. Yet none of these studies in transdiagnostic clinical settings reported follow-up data on psychological flexibility so far. Nor did either of these studies examine the impact of altered flexibility on quality of life, although this is the stated primary goal of ACT. The present study aims to help fill these gaps by examining the following questions: Does psychological flexibility change during and after a treatment in a transdiagnostic psychiatric day hospital? And how is the change related to quality of life, general functioning, and symptoms?

## 2 Methods

### 2.1 Procedure

The above questions were investigated as part of a larger effectiveness study (26). The investigation was carried out in accordance with the Declaration of Helsinki (2013). The research project was approved by the Ethics Committee of the Medical Association Berlin (12th February 2020, case number Eth-03/20) and was retrospectively registered in the German Clinical Trials Register (http://www.drks.de/DRKS00029992, identifier: DRKS00029992) on August 19th, 2022. Participants were recruited in a psychiatric day hospital in Berlin, Germany. All participants in the study gave their written informed consent.

### 2.2 Participants

92 participants were included in the evaluation trial. For a detailed flow of participants, s. Rutschmann et al. (26). Two participants did not respond to the Acceptance and Action Questionnaire (AAQ-II) and were therefore excluded from statistical analyses for the present study. Of the 90 participants included in the present study, 47 participated at all three survey time points. The remaining participants participated in the pre- and post-treatment survey or in the pre-treatment and follow-up survey. They were also included in the statistical analyses.

The participants were between 18 and 65 years old (Mdn = 39.5) and the majority (64.4%) identified themselves as female (due to standardized questionnaires, it was only possible to decide between male and female). The main diagnoses were mood disorders (58.9%), anxiety disorders (13.3%), reaction to severe stress, and adjustment disorders (10.0%), psychotic disorders (8.9%), personality disorders (5.6%), and somatoform disorders (3.3%). The comorbidity rate was 46.7%, and 10.0% of the participants had more than two psychiatric diagnoses.

### 2.3 Treatment

ACT had been implemented in the psychiatric day hospital for approximately one year before the start of the study. The entire professional team, including physicians, psychologists, nurses, social workers, movement therapists, and music therapists, had been trained in ACT and participated regularly in ACT-supervision sessions.

The therapy focus was on promoting psychological flexibility in transdiagnostic group sessions, including ACT group psychotherapy twice a week for 50 minutes each, based on the Wengenroth (27) material, and occupational therapy, art therapy, movement therapy, mindfulness training and an ACT-Matrix group about once a week for 50 minutes each (cf. 26). The ACT-Matrix is a tool to distinguish between internal vs external events on the one hand, and approach vs avoidance behaviors on the other hand, thus supporting value-oriented, flexible behavior (28). In addition, regular ACT-based one-on-one therapy was offered to address individual issues and problems once a week for 25 minutes. Each week, a different one of the six core processes of psychological flexibility was focused on across groups and professions. So, if, for example, the focus was on the core process of acceptance in one week, this was not only treated in an experience-oriented way in the ACT group twice this week, but also in art therapy, movement therapy etc. The regular length of stay was at least six weeks to ensure that each core process was completed once.

Treatment conditions had to be adjusted due to the pandemic, but it was always ensured that ACT group therapy and ACT-based individual therapy took place as described above.

### 2.4 Measures

All patients received a set of questionnaires at admission, discharge, and 3 months after discharge. At admission and discharge, the therapist noted the diagnosis after an unstandardized interview based on clinical impression. Additionally, the therapist assessed the current level of functioning using the Global Assessment of Functioning Scale (GAF) (29).

Psychological flexibility was assessed with the AAQ-II (30). The AAQ-II consists of 7 items, each to be answered on a 7-point Likert-scale. The sum score indicates the degree of psychological flexibility: The higher the sum score, the lower the flexibility. The AAQ-II is recognized as a unidimensional, reliable, and valid instrument for assessing psychological flexibility (20, 30, 31). It is unspecific for diagnoses and can be used universally (20). Translations into many languages exist, as well as specific AAQ questionnaires for different diagnoses (32).

Symptom severity was assessed using the Global Severity Index (GSI) of the Symptom Checklist-90-Standard (SCL-90-S) (33). The SCL-90-S was also used to assess the level of education and the gender.

The depressed mood was assessed using the sum score of the Beck Depression Inventory II (BDI-II) (34).

The World Health Organization Quality of Life—Short Version (WHOQOL-BREF) (35) was used to measure subjective quality of life in the dimensions of physical and psychological well-being, social relationships, environment, and global quality of life.

After discharge, it was also noted whether the medication was administered during the stay, based on clinical guidelines, with adaptations as necessary (applied/increased, switched, decreased/stopped, or left unchanged).

### 2.5 Data analyses

Differences between the final sample and dropouts regarding the AAQ-II were analyzed via two-sided independent *t*-tests (IBM SPSS Statistics Version 28.0.1.0). Missing data of all studied variables were analyzed by Little’s MCAR test. Pre- to post-, pre- to follow-up- and post- to follow-up-differences in psychological flexibility were tested using two-sided paired samples *t*-tests.

Correlations of residualized changes were calculated to identify associations between changes in psychological flexibility and changes in quality of life, symptom severity, depressed mood, and global functioning respectively. The threshold of significance was adjusted according to the Bonferroni method to *p* <.006 for correlations of pre- to post-changes and to *p* <.007 for correlations of pre- to follow-up-changes.

Possible associations between individual characteristics and psychological flexibility at the time of admission were examined via Pearson correlation (age), point-biserial correlations (gender) and ANOVA (educational level, main diagnosis).

In addition, possible individual factors influencing changes in psychological flexibility were examined, using Pearson correlation (age), point-biserial correlations (gender), and ANOVA (educational level, main diagnosis, change of medication).

## 3 Results

The final sample and the dropouts did not differ regarding their AAQ-II sum at any survey time (all *p* >.23). Missing data in the final sample were missing completely at random (Little’s MCAR test, *p* = .47), justifying the use of pairwise deletion for the following analyses. This leads to varying sample sizes in the different analyses.

Psychological flexibility, as measured via the AAQ-II, increased significantly between admission and discharge (*t*(84) = 3.96, *p* <.001, *d* = .43), and between admission and follow-up (*t*(51) = 3.92, *p <*.001, *d* = .54). There was no significant difference in psychological flexibility between discharge and follow-up (*t*(46) = 0.47, *p* = .64, *d* = .07). A similar pattern was found for the other outcome measures (BDI-II, WHOQOL-BREF, GAF, GSI). All of them improved between admission and discharge, and the effects remained stable, with no significant changes between discharge and follow-up. Tables with detailed information on this can be found in the larger effectiveness study (26).

There was no significant correlation between psychological flexibility at admission and age or gender (all *p* >.13). The ANOVA showed no significant difference in psychological flexibility between different levels of education (*p* = .26). The ANOVA showed a significant difference in psychological flexibility between different main diagnoses (*p* = .002): Schizophrenia, schizotypal, and delusional disorders (*M* = 19.56, *SD* = 8.65), affective disorders (*M* = 30.72, *SD* = 9.00), neurotic, stress-related, and somatoform disorders (*M* = 27.96, *SD* = 9.89), and personality disorders (*M* = 38.00, *SD* = 2.55).

Residualized changes in psychological flexibility correlated significantly with residualized changes in symptom severity, depressed mood, global functioning, and most measures of quality of life (see **Table 1**). This was true both for changes between admission and discharge and between admission and follow-up. The correlation between the residualized change scores of psychological flexibility and quality of life in the environment dimension failed to reach significance in the pre-treatment to post-treatment comparison (*p* = .009) but was significant in the pre-treatment to follow-up-comparison. The effect sizes of the correlations were generally larger for the pre-treatment to follow-up comparisons.

**Table 1:** Bivariate correlations between residualized change scores of the AAQ-II and other study measures.

*Variable*	Pre-to Post-Treatment				Pre-Treatment to Follow-Up			
	n	*r*		*p*	n	*r*		*p*
BDI	78	.60	**	<.001	51	.83	**	<.001
GSI	78	.60	**	<.001	51	.75	**	<.001
GAF	76	-.34	*	.003	–	–		–
QL								
psy.	81	-.61	**	<.001	52	-.79	**	<.001
soc.	79	-.43	**	<.001	51	-.68	**	<.001
phys.	79	-.47	**	<.001	50	-.75	**	<.001
env.	81	-.29		.009	52	-.47	**	<.001
glob.	79	-.52	**	<.001	51	-.66	**	<.001

Higher values in AAQ-II imply lower psychological flexibility. Therefore, negative correlations indicate a positive association between the study measure and psychological flexibility and vice versa. *p < .006 (pre- to post-treatment) or p < .007 (pre-treatment to follow-up), **p < .001 , "-" means not "applicable".

Residualized pre-treatment to follow-up changes in psychological flexibility correlated significantly with age (*r* = .43, *p* = .002), with older participants showing smaller lasting changes. Apart from that, there were no other significant associations between participant variables (gender, educational level, main diagnosis, change of medication) and changes in psychological flexibility (all *p* >.13).

## 4 Discussion

### 4.1 Key findings and interpretation

ACT considers psychological flexibility an important transdiagnostic factor in the pathogenesis of mental disorders. This view has been supported by the findings of several meta-analyses that found associations between psychological flexibility and various measures of symptomatology, stress, pain, and quality of life (12–14). Increasing psychological flexibility is therefore seen as an important mechanism of change in ACT-based therapies. Consequently, psychological flexibility has also been repeatedly examined as a process variable of therapeutic change in studies on ACT.

However, although ACT is considered a transdiagnostic therapy method, it has not been studied in transdiagnostic clinical settings for a long time. As far as we know, there have been only three other studies on ACT in such settings so far (23–25). Additionally, no study in a transdiagnostic clinical setting has yet reported longitudinal data on psychological flexibility as well as associations between changes in psychological flexibility and changes in quality of life.

This study examined changes of psychological flexibility during and three months after a treatment in an ACT-based transdiagnostic day clinic and their association with changes in symptoms, quality of life, and general functioning. Participants showed higher levels of psychological flexibility at discharge and three months after treatment compared to admission. This change was associated with improved quality of life, reduced symptom burden, and improved general functioning. These findings are consistent with other studies that have found an association between increased psychological flexibility and reduced symptom burden following a transdiagnostic clinical treatment (23–25). In addition, results from other settings could be replicated that an increase in psychological flexibility is associated with a higher quality of life and level of functioning (12, 13).

Interestingly, the correlations between psychological flexibility and symptoms as well as quality of life were stronger in the follow-up than in the post-treatment, while none of these variables themselves changed significantly between post-treatment and follow-up (s. 26). One possible explanation could be that patients, who have sustained increases in psychological flexibility, may benefit more in the long term, whereas outcomes at discharge may be more influenced by additional factors of the treatment (such as day structure and social contacts in the day hospital).

Another interesting side fact is that, while age was not significantly correlated to psychological flexibility at admission, older patients showed lower lasting changes in psychological flexibility three months after the treatment. This could be due to the age-related cognitive decline in learning ability (36). One conclusion of that might be that older people need longer or more intensive training in ACT to persistently increase their psychological flexibility and thus benefit persistently. On the other hand, ACT has shown to be a promising therapy method for the elderly (37). Additionally, a review has shown older patients to have a higher average psychological flexibility on other measures than on the AAQ-II (37). Although psychological flexibility at admission differed significantly by diagnosis, there was no difference in change in psychological flexibility during treatment. This supports the view of psychological flexibility as a transdiagnostic factor in therapy.

### 4.2 Limitations and future directions

The most important limitation arises from the naturalistic study design. Statements about causality are only possible in experimental designs. Therefore, we can only describe which phenomena occur together, not in which causal relationship they stand. The lack of a control group and repeated testing during treatment also do not allow conclusions about mediation effects (cf. 38).

Another potential limitation arises from the use of the AAQ-II. In recent years, there has been increasing disagreement about what the AAQ-II actually measures (39–41). Some of the confusion can be attributed to the inconsistent use of terms in the literature. Some authors refer to the AAQ-II as a measure of psychological flexibility or inflexibility, others as a measure of experiential avoidance, and still others as a measure of acceptance (41). The distinction is important though because, strictly speaking, the antipoles acceptance and experiential avoidance represent only one of the six core processes of psychological flexibility (cf. above). Some authors suggested that the AAQ-II may rather measure negative emotionality than psychological flexibility (39, 42). On the other hand, previous examinations have proven the AAQ-II to have incremental utility above neuroticism or depressive and anxiety symptomology (20).

The use of the term psychological flexibility in connection with the AAQ-II has also been criticized. The AAQ-II was designed to measure psychological inflexibility and it remains questionable whether the absence of inflexibility implies more flexibility, or whether non-inflexibility is equivalent to flexibility (40, 41). For reasons of better readability and on the basis of the term used in the literature, we are still referring to flexibility here as well. Assuming that a reduction in inflexibility is at least accompanied by an increase in flexibility, we are referring to flexibility here, nevertheless, this point has to be considered as a potential limitation.

Another point of criticism is that the AAQ-II measures psychological flexibility as a unidimensional construct, while the underlying theory is that psychological flexibility consists of six interrelated core processes (40, 41, 43–45). On the other hand, it has been argued, that psychological flexibility is a higher level construct, rather than simply the sum of the six core processes (20). In response to the various criticisms of the AAQ-II, several other questionnaires have been developed in recent years to assess psychological flexibility, such as the Open and Engagement State Questionnaire OESQ (46), the Multidimensional Psychological Flexibility Index MPFI (44) or the Psy-Flex (47). All of these measures require further validation to test whether they are preferable to the AAQ-II. Until now, the AAQ-II remains the most frequently used and best studied measure for psychological flexibility (41).

This study makes some important contributions to the research on psychological flexibility as a process variable in transdiagnostic treatments. It is one of the first to examine changes in psychological flexibility in a transdiagnostic clinical setting. It is the first in a transdiagnostic clinical setting that examines the associations between changes in psychological flexibility and quality of life. And it is the first in such a setting to report longitudinal data of psychological flexibility.

To investigate the hypothesis of psychological flexibility as a process variable in transdiagnostic ACT treatment, further studies are needed. The use of a control group, as well as repeated measurements in the process, would be necessary to make sound statements about mediation. In addition, longer follow-up intervals should be used to investigate the role of lasting changes in psychological flexibility and their association with symptoms and quality of life. The role of age and its possible effects on ACT and psychological flexibility should also be investigated in future studies.

## Data availability statement

The raw data supporting the conclusions of this article will be made available by the authors, without undue reservation.

## Ethics statement

The studies involving humans were approved by Ethics Committee of the Medical Association Berlin. The studies were conducted in accordance with the local legislation and institutional requirements. The participants provided their written informed consent to participate in this study.

## Author contributions

RR: Conceptualization, Data curation, Formal analysis, Investigation, Methodology, Visualization, Writing – original draft, Writing – review & editing. NR: Conceptualization, Funding acquisition, Supervision, Writing – review & editing. AG: Conceptualization, Supervision, Writing – review & editing. CR: Conceptualization, Funding acquisition, Methodology, Resources, Supervision, Writing – review & editing.
